# Impact of Larger Sputum Volume on Xpert^®^ MTB/RIF Assay Detection of *Mycobacterium tuberculosis* in Smear-Negative Individuals with Suspected Tuberculosis

**DOI:** 10.3390/jcm6080078

**Published:** 2017-08-07

**Authors:** Sharlaa Badal-Faesen, Cynthia Firnhaber, Michelle A. Kendall, Xingye Wu, Beatriz Grinsztejn, Rodrigo Otavio da Silva Escada, Michel Fernandez, Evelyn Hogg, Ian Sanne, Pamela Johnson, David Alland, Gerald H. Mazurek, Debra A. Benator, Anne F. Luetkemeyer, the AIDS Clinical Trials Group A5295, Tuberculosis Trials Consortium Study 34 Teams

**Affiliations:** 1 Clinical HIV Research Unit, Department of Internal Medicine, School of Clinical Medicine, Faculty of Health Sciences, University of Witwatersrand, Johannesburg 2092, South Africa; csfirnhaber@gmail.com (C.F.); isanne@witshealth.co.za (I.S.); 2 Center for Biostatistics in AIDS Research, Harvard T.H. Chan School of Public Health Boston, Boston, MA 02115, USA; kendall@sdac.harvard.edu (M.A.K.); xwu@sdac.harvard.edu (X.W.); 3 Instituto Nacional de Infectologia Evandro Chagas-INI-Fiocruz, Rio de Janeiro 21.045-900, Brazil; gbeatriz@ini.fiocruz.br (B.G.); rodrigo.escada@ini.fiocruz.br (R.O.S.E.); 4 Pulmonary and Critical Care Department, Baylor Scott and White Hospital, Temple, TX 76508, USA; michel_6815@yahoo.com; 5 Social & Scientific Systems, Inc., Silver Spring, MD 20910, USA; ehogg@s-3.com; 6 Cepheid Inc., Sunnyvale, CA 94089, USA; pamela.johnson@cepheid.com; 7 Division of Infectious Disease, Rutgers-New Jersey Medical School, 185 South Orange Avenue, Newark, NJ 07103, USA; allandda@njms.rutgers.edu; 8 Division of Tuberculosis Elimination, Centers for Disease Control and Prevention, Atlanta, GA 30333, USA; gym6@cdc.gov; 9 The Veterans Affairs Medical Center, The George Washington University, Washington, DC 20422, USA; debra.benator@va.gov; 10 Division of HIV, Infectious Diseases and Global Medicine, Zuckerberg San Francisco General, University of California, San Francisco, CA 94110, USA

**Keywords:** larger volume sputum, Xpert MTB/RIF, smear-negative suspects

## Abstract

As a strategy to improve the sensitivity of nucleic acid-based testing in acid-fast bacilli (AFB) negative samples, larger volumes of sputum (5–10 mL) were tested with Xpert^®^ MTB/RIF from 176 individuals with smear-negative sputum undergoing tuberculosis evaluation. Despite larger volumes, this strategy had a suboptimal sensitivity of 50% (4/8).

## 1. Introduction 

According to the World Health Organization, 2015 Global Tuberculosis (TB) report [1], 9.6 million people developed TB in 2014. Diagnosing TB is challenging, particularly in people with Human Immunodeficiency Virus (HIV) infection who are more likely to have paucibacillary pulmonary disease and in whom tuberculosis causes significant morbidity and mortality. 

Xpert^®^ MTB/RIF Assay (Xpert) is more sensitive in identifying *Mycobacterium tuberculosis* (*Mtb*) than traditional acid-fast bacilli (AFB) smear, as Xpert requires only five genome copies of purified DNA per reaction or 131 colony forming units per mL of sputum to identify *Mtb*, whereas 10,000 bacilli per mL of sputum are required for detection by AFB smear [2]. However, Xpert MTB/RIF using the standard sputum volume of 1 to 3 mL, either as unprocessed sputum or as a subsequently concentrated pellet, missed AFB smear-negative disease, with 1 Xpert identifying 59% and 2 Xperts identifying 71% of individuals with culture-positive but smear-negative pulmonary TB [3].

We hypothesized that larger volumes (LV) of sputum (5–10 mL) would contain more *Mtb* and may improve the diagnostic yield for TB using Xpert, especially in people with paucibacillary pulmonary TB. LV sputum has been shown to improve the detection of AFB by smear [4,5,6]. LV sputum may be a practical alternative to repeated sputum collections, particularly in rural areas [6]. 

## 2. Methods

*Participants*: Adults enrolled in a larger evaluation of Xpert conducted by the Acquired Immunodeficiency Syndrome (AIDS) Clinical Trials Group and the Tuberculosis Trials Consortium (TBTC) were asked to provide an LV sputum sample in addition to the standard volume sputum samples provided for the parent study. The parent study has been published [3]. Adults undergoing evaluation for pulmonary TB at 21 sites in the US, Rio de Janeiro, Brazil, and Johannesburg, South Africa were asked to participate in the parent study if they had received ≤48 h of TB treatment in the six months prior to sputum collection. This sub-study was limited to AFB smear-negative participants who provided an LV sputum sample.

*Larger volume sputum collection and processing*: LV sputa were collected by pooling sputa over several hours or overnight, and most were collected after at least one standard volume sputum was obtained. The LV specimen was digested and decontaminated using 1% NALC/NaOH [7] and centrifuged into a pellet prior to Xpert testing and resuspended with sample reagent in a 3:1 ratio. Neither AFB smear nor culture was performed on these specimens, as the entire sputum volume was used for Xpert testing to produce the highest possible yield. AFB smear and culture was performed on the accompanying sputum specimens.

*Statistical Methods*: AFB smear-negative status was defined as neither of the two sputum specimens collected for the parent study being positive by fluorescent staining. Culture-confirmed TB was defined as at least one of the four cultures (two sputum specimens, each cultured on liquid and solid media) presenting *Mtb* growth. Sensitivity and specificity were calculated for standard volume and LV Xpert results with 95% confidence intervals (CIs) using Wilson’s score binomial method. The gold standard comparator for *Mtb* detection was culture-confirmed TB. Xpert results from the first standard volume sputum and the LV sputum were compared. Within-participant comparison of sensitivities was assessed using McNemar’s mid-p test. 

*Ethics*: The protocol was approved by the institutional review board/ethics committee at each site and the Centers for Disease Control and Prevention. 

## 3. Results

*Population*: Of the 992 participants in the parent study, 838 (84%) participants were AFB smear-negative, and 21% (176/838) of the smear-negative participants provided an LV sputum specimen. Median age was 45 years (*Q1, Q3* = 36, 53). Sixty-two percent (109/176) were from Brazil, 38% (66/176) from the US, and <1% (1/176) from South Africa (where fewer smear-negative participants provided LV specimens). Thirty-four percent (59/176) had HIV infection. Six percent (11/176) had a history of prior TB and 9% (16/176) had initiated TB treatment. Ninety-eight percent (173/176) had symptoms of TB. Median time for LV collection was 0.02 h (*Q1, Q3* = 0.2, 0.85), with a minority (13%) requiring ≥3 h for collection and 14 individuals requiring overnight collection. 

*Microbiologic diagnoses*: Among the 176 participants, eight (5%) had culture-confirmed TB, 165 (94%) had no *Mtb* growth on any culture, and three (2%) had contamination on all cultures (i.e., bacterial overgrowth preventing AFB detection).

*Xpert Performance*: Of the 176 LV sputa, Xpert was positive for five (3%), negative for 162 (92%), failed for six (3%), and not done for three (2%) due to site or laboratory error. Of note, the failure rate for the first Xpert test in the parent study was 2% [3]. Among the eight participants with culture-confirmed TB, Xpert on the LV sputa identified four (50.0%) while Xpert on the standard volume sputum identified one (12.5%) (McNemar’s mid-p *p* = 0.125). There were two Xpert false positives for MTB, one standard and one LV, occurring in different participants. As shown in Table 1, specificities for Xpert using LV sputa and standard volume sputa were equivalent (99.4%).

## 4. Discussion

LV sputum was associated with increased yield compared to standard volume Xpert testing. However, this increase was not statistically significant, which may be due in part to the small number of smear-negative participants who provided LV samples and the rarity of culture-confirmed TB in this sub-study. Moreover, large volume Xpert testing sensitivity remained suboptimal at 50.0%, similar to the sensitivity of a single standard volume Xpert testing on a smear-negative specimen. This strategy merits further investigation, as an intervention that potentially could increase the yield of Xpert testing in sputum-smear-negative TB disease, which is important because a single Xpert test on standard volume sputum may miss a substantial number of culture-confirmed smear-negative pulmonary TB cases [3,8]. Repeating Xpert testing can increase diagnostic yield, but imposes inconvenience and cost [9]. Xpert testing of LV sputum requires additional collection time for the patient, an additional step of centrifugation prior to testing, and possibly another clinic visit; this may limit the utility of the LV strategy in some resource-limited settings. The Xpert “Ultra”, currently under development, is expected to improve the ability of a single Xpert to identify TB in smear-negative individuals with sensitivity similar to mycobacterial culture [10]. Until this assay is available, further investigation of Xpert on LV sputum specimens may be warranted. 

## Figures and Tables

**Table 1:** Xpert Sensitivity and Specificity Results.

Sputum Volume	Sensitivity	Specificity *	PPV	NPV
Standard Volume Xpert ^†^ (*n* = 172)	12.5% (1/8)	99.4% (163/164)	50.0% (1/2)	95.9% (163/170)
	[2.2%, 47.1%]	[96.6%, 99.9%]	[9.5%, 90.5%]	[91.7%, 98.0%]
Large Volume Xpert ^‡^ (*n* = 164)	50.0% (4/8)	99.4% (155/156)	80.0% (4/5)	97.5% (156/159)
	[21.5%, 78.5%]	[96.5%, 99.9%]	[37.6%, 96.4%]	[93.7%, 99.0%]

Data summarized as % (*n*/*n*) [95% CIs]; PPV = Positive Predictive Value; NPV = Negative Predictive Value. * If limited to the 155 participants with both standard volume and large volume Xpert results, specificity was the same for standard volume and large volume Xpert: 99.4% (154/155) [96.4%, 99.9%]; ^†^ Four participants were excluded due to one Xpert failure using standard volume sputum and contamination of all cultures for three participants; ^‡^ Twelve participants were excluded due to six Xpert failures using large volume sputa, three site errors leading to no large volume Xpert results, and contamination of all cultures for three participants.
